# Supplementary figures and images for: H3K4 Methylation Dependent and Independent Chromatin Regulation by JHD2 and SET1 in Budding Yeast
Source: G3 (Bethesda). 2018 Mar 29;8(5):1829–39. doi: 10.1534/g3.118.200151 (PMC5940172; doi:10.1534/g3.118.200151)

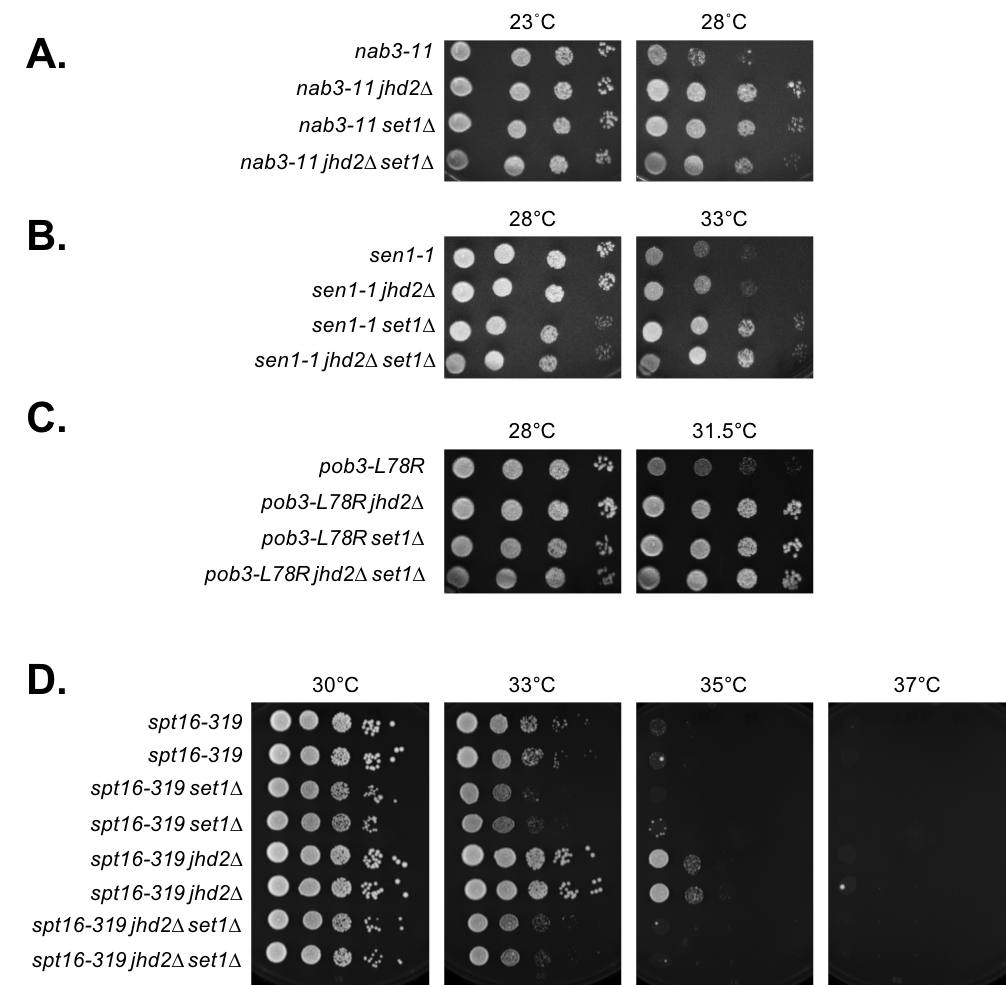

Supplement: Supplementary file 1 [file 1829FigureS1.tif]

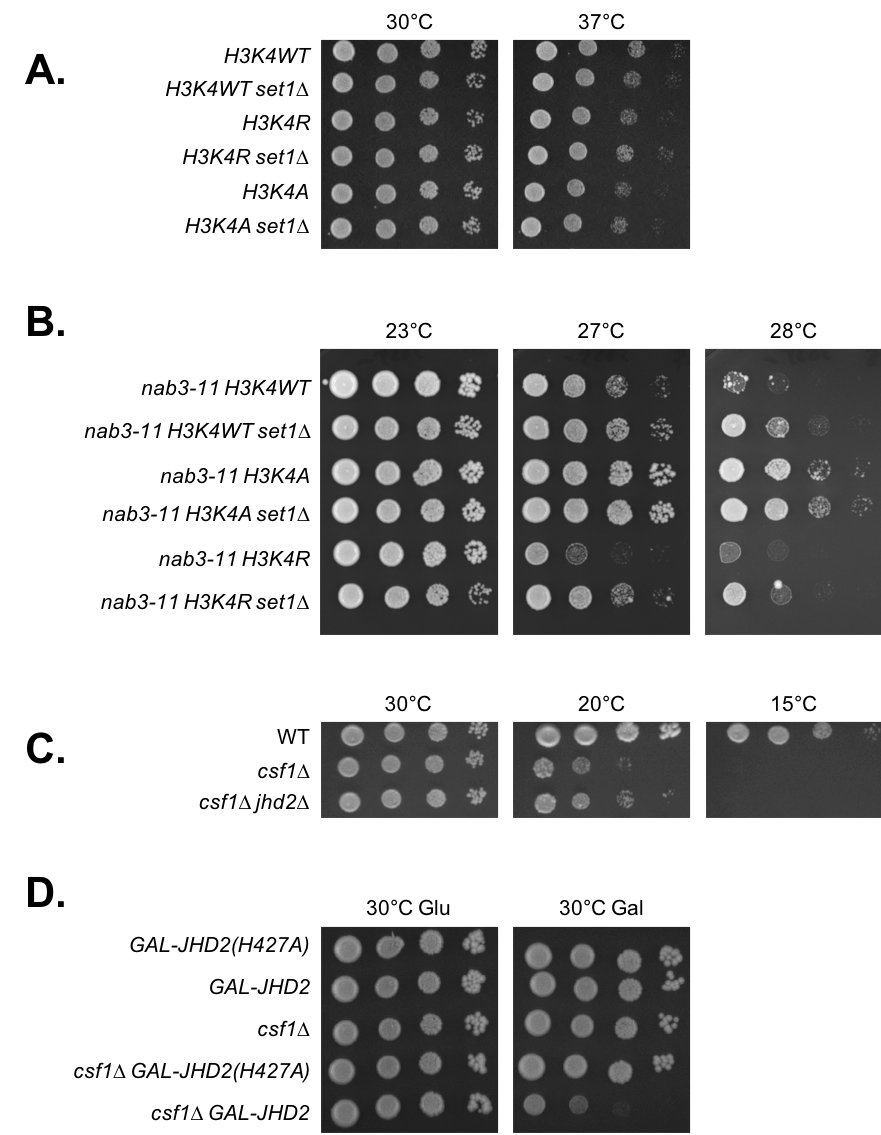

Supplement: Supplementary file 2 [file 1829FigureS2.tif]

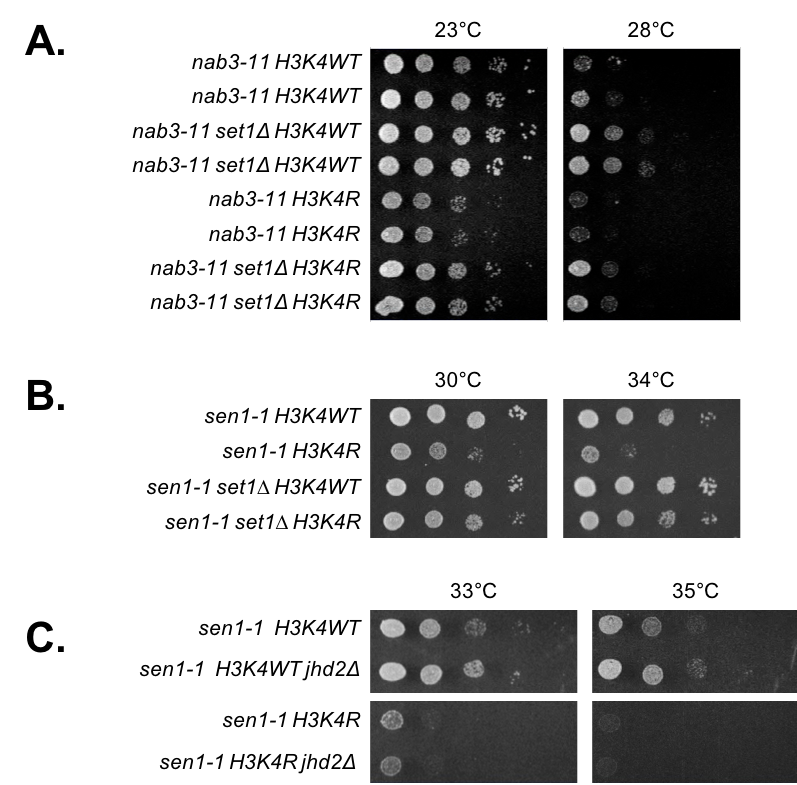

Supplement: Supplementary file 3 [file 1829FigureS3.tif]

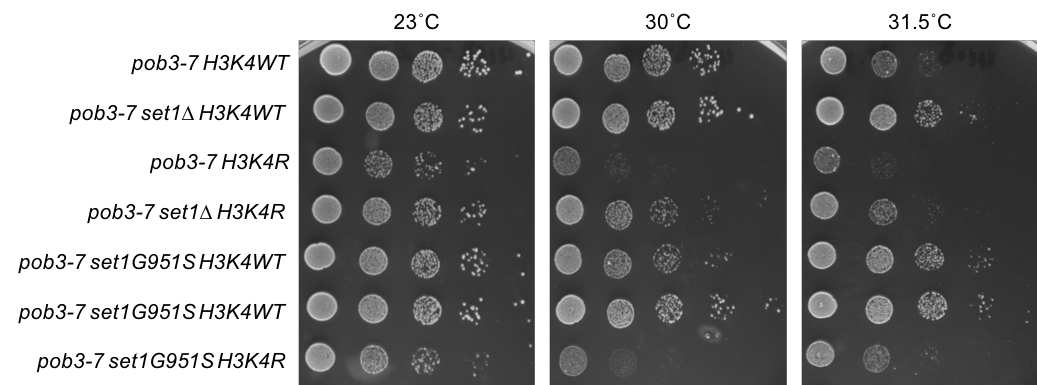

Supplement: Supplementary file 4 [file 1829FigureS4.tif]

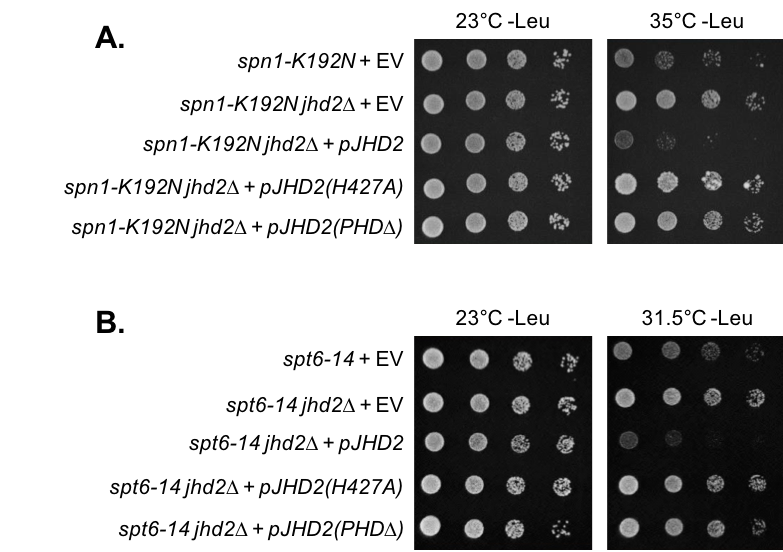

Supplement: Supplementary file 5 [file 1829FigureS5.tif]

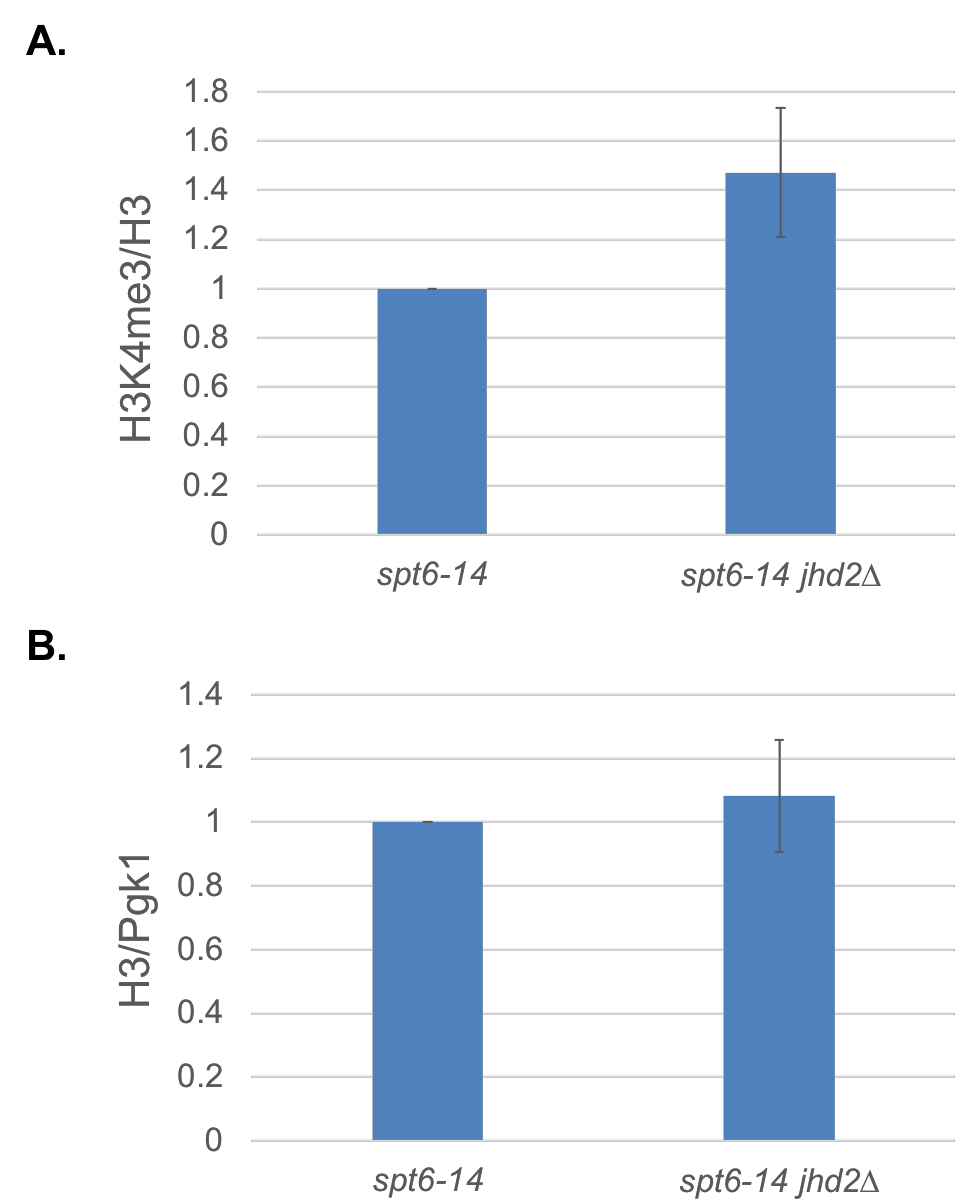

Supplement: Supplementary file 6 [file 1829FigureS6.tif]
