# Supplementary material for: H3K4 Methylation Dependent and Independent Chromatin Regulation by JHD2 and SET1 in Budding Yeast
Source: G3 (Bethesda). 2018 Mar 29;8(5):1829–39. doi: 10.1534/g3.118.200151 (PMC5940172; doi:10.1534/g3.118.200151)
Supplement: Supplementary file 7 [file 1829TableS1.docx]

| **Strain Name** | Mating Type | Genotype |  |
| --- | --- | --- | --- |
| kly2172 | Mat**α** | MET15+ LYS2+ his3 leu2 ura3 WT |  |
|  |  | unless otherwise indicated, all strains are MET15+, LYS2+, his3, leu2, ura3 |  |
| kly1316 | Mat**a** | set1∆::natMX6 |  |
| kly1319 | Mat**a** | set1∆::natMX6 jhd2∆::hygMX6 |  |
| kly1693 | Mat**a** | spt16-ts::kanMX4 |  |
| kly1694 | Mat**a** | spt16-ts::kanMX4 jhd2∆::hygMX6 |  |
| kly1697 | Mat**a** | spt16-ts::kanMX4 set1∆::natMX6 |  |
| kly1698 | Mat**a** | spt16-ts::kanMX4 set1∆::natMX6 jhd2∆::hygMX6 |  |
| kly1742 | Mat**α** | pob3-7::kanMX4 hht1-hhf1∆::natMX4 hht2-hhf2::[HHTS-HHFS]H3K4WT-URA3 |  |
| kly1743 | Mat**a** | pob3-7::kanMX4 hht1-hhf1∆::natMX4 hht2-hhf2::[HHTS-HHFS]H3K4WT-URA3 jhd2∆::hygMX6 |  |
| kly1748 | Mat**α** | pob3-7::kanMX4 hht1-hhf1∆::natMX4 hht2-hhf2::[HHTS-HHFS]H3K4R-URA3 |  |
| kly1749 | Mat**a** | pob3-7::kanMX4 hht1-hhf1∆::natMX4 hht2-hhf2::[HHTS-HHFS]H3K4R-URA3 jhd2∆::hygMX6 |  |
| kly1909 | Mat**α** | spn1-K192N::kanMX4 bre2∆::natMX6 |  |
| kly1910 | Mat**α** | spn1-K192N::kanMX4 bre2∆::natMX6 jhd2∆::hygMX6 |  |
| kly2053 | Mat**α** | jhd2∆::hygMX6 |  |
| kly2095 | Mat**α** | spt6-14::kanMX4 |  |
| kly2096 | Mat**a** | spt6-14::kanMX4 jhd2∆::hygMX6 |  |
| kly2097 | Mat**α** | spt6-14::kanMX4 set1∆::natMX6 |  |
| kly2098 | Mat**α** | spt6-14::kanMX4 set1∆::natMX6 jhd2∆::hygMX6 |  |
| kly2139 | Mat**α** | spn1-K192N::kanMX4 spp1∆::natMX6 |  |
| kly2140 | Mat**a** | spn1-K192N::kanMX4 spp1∆::natMX6 jhd2∆::hygMX6 |  |
| kly2478 | Mat**α** | spn1-K192N::kanMX4 hht1-hhf1∆::natMX4 hht2-hhf2::[HHTS-HHFS]H3K4WT-URA3 |  |
| kly3260 | Mat**α** | spn1-K192N::kanMX4 hht1-hhf1∆::natMX4 hht2-hhf2::[HHTS-HHFS]H3K4R-URA3 |  |
| kly3261 | Mat**a** | spn1-K192N::kanMX4 hht1-hhf1∆::natMX4 hht2-hhf2::[HHTS-HHFS]H3K4R-URA3 jhd2∆::hygMX6 |  |
| kly3532 | Mat**α** | nab3-42::URA3 set1∆::natMX6 |  |
| kly3534 | Mat**α** | nab3-42::URA3 jhd2∆::hygMX6 |  |
| kly3536 | Mat**α** | nab3-42::URA3 set1∆::natMX6 jhd2∆::hygMX6 |  |
| kly3707 | Mat**α** | sen1-1::kanMX4 hht1-hhf1∆::natMX4 hht2-hhf2::[HHTS-HHFS]H3K4WT-URA3 |  |
| kly3708 | Mat**a** | sen1-1::kanMX4 hht1-hhf1∆::natMX4 hht2-hhf2::[HHTS-HHFS]H3K4WT-URA3 jhd2∆::hygMX6 |  |
| kly3726 | Mat**a** | spn1-K192N::kanMX4 hht1-hhf1∆::natMX4 hht2-hhf2::[HHTS-HHFS]H3K4WT-URA3 jhd2∆::hygMX6 |  |
| kly3729 | Mat**α** | sen1-1::kanMX4 hht1-hhf1∆::natMX4 hht2-hhf2::[HHTS-HHFS]H3K4R-URA3 |  |
| kly3730 | Mat**a** | sen1-1::kanMX4 hht1-hhf1∆::natMX4 hht2-hhf2::[HHTS-HHFS]H3K4R-URA3 jhd2∆::hygMX6 |  |
| kly3844 | Mat**α** | nab3-11::kanMX4 |  |
| kly3845 | Mat**a** | nab3-11::kanMX4 jhd2∆::hygMX6 |  |
| kly3855 | Mat**a** | nab3-11::kanMX4 hht1-hhf1∆::natMX4 hht2-hhf2::[HHTS-HHFS]H3K4WT-URA3 |  |
| kly3856 | Mat**α** | nab3-11::kanMX4 hht1-hhf1∆::natMX4 hht2-hhf2::[HHTS-HHFS]H3K4WT-URA3 |  |
| kly3857 | Mat**a** | nab3-11::kanMX4 hht1-hhf1∆::natMX4 hht2-hhf2::[HHTS-HHFS]H3K4WT-URA3 set1∆::hygMX6 |  |
| kly3858 | Mat**a** | nab3-11::kanMX4 hht1-hhf1∆::natMX4 hht2-hhf2::[HHTS-HHFS]H3K4WT-URA3 set1∆::hygMX6 |  |
| kly3863 | Mat**a** | nab3-11::kanMX4 hht1-hhf1∆::natMX4 hht2-hhf2::[HHTS-HHFS]H3K4R-URA3 |  |
| kly3864 | Mat**α** | nab3-11::kanMX4 hht1-hhf1∆::natMX4 hht2-hhf2::[HHTS-HHFS]H3K4R-URA3 |  |
| kly3865 | Mat**a** | nab3-11::kanMX4 hht1-hhf1∆::natMX4 hht2-hhf2::[HHTS-HHFS]H3K4R-URA3 set1∆::hygMX6 |  |
| kly3866 | Mat**a** | nab3-11::kanMX4 hht1-hhf1∆::natMX4 hht2-hhf2::[HHTS-HHFS]H3K4R-URA3 set1∆::hygMX6 |  |
| kly4068 | Mat**α** | spn1-K192N::kanMX4 set1∆::natMX6 jhd2∆::hygMX6 |  |
| kly4069 | Mat**α** | spn1-K192N::kanMX4 set1∆::natMX6 |  |
| kly4070 | Mat**a** | spn1-K192N::kanMX4 jhd2∆::hygMX6 |  |
| kly4071 | Mat**a** | spn1-K192N::kanMX4 |  |
| kly4105 | Mat**α** | pob3-7::kanMX4 hht1-hhf1∆::natMX4 hht2-hhf2::[HHTS-HHFS]H3K4R-URA3 set1G951S |  |
| kly4108 | Mat**α** | pob3-7::kanMX4 hht1-hhf1∆::natMX4 hht2-hhf2::[HHTS-HHFS]H3K4WT-URA3 set1G951S |  |
| kly4109 | Mat**α** | pob3-7::kanMX4 hht1-hhf1∆::natMX4 hht2-hhf2::[HHTS-HHFS]H3K4WT-URA3 set1G951S |  |
| kly4155 | Mat**a** | pob3-7::kanMX4 hht1-hhf1∆::natMX4 hht2-hhf2::[HHTS-HHFS]H3K4A-URA3 |  |
| kly4157 | Mat**α** | pob3-7::kanMX4 hht1-hhf1∆::natMX4 hht2-hhf2::[HHTS-HHFS]H3K4A-URA3 set1∆::hygMX6 |  |
| kly4163 | Mat**a** | pob3-7::kanMX4 hht1-hhf1∆::natMX4 hht2-hhf2::[HHTS-HHFS]H3K4WT-URA3 |  |
| kly4165 | Mat**α** | pob3-7::kanMX4 hht1-hhf1∆::natMX4 hht2-hhf2::[HHTS-HHFS]H3K4WT-URA3 set1∆::hygMX6 |  |
| kly4167 | Mat**α** | pob3-7::kanMX4 hht1-hhf1∆::natMX4 hht2-hhf2::[HHTS-HHFS]H3K4R-URA3 |  |
| kly4169 | Mat**α** | pob3-7::kanMX4 hht1-hhf1∆::natMX4 hht2-hhf2::[HHTS-HHFS]H3K4R-URA3 set1∆::hygMX6 |  |
| kly4183 | Mat**a** | spt16-319::natMX6 |  |
| kly4184 | Mat**α** | spt16-319::natMX6 |  |
| kly4185 | Mat**a** | spt16-319::natMX6 set1∆::hygMX6 |  |
| kly4186 | Mat**α** | ura3 spt16-319::natMX6 set1∆::hygMX6 |  |
| kly4187 | Mat**a** | spt16-319::natMX6 jhd2∆::kanMX4 |  |
| kly4188 | Mat**α** | spt16-319::natMX6 jhd2∆::kanMX4 |  |
| kly4189 | Mat**a** | spt16-319::natMX6 set1∆::hygMX6 jhd2∆::kanMX4 |  |
| kly4190 | Mat**α** | spt16-319::natMX6 set1∆::hygMX6 jhd2∆::kanMX4 |  |
| mmy3877 | Mat**α** | pob3-7::kanMX4 |  |
| mmy3878 | Mat**α** | pob3-7::kanMX4 set1∆::natMX6 jhd2∆::hygMX6 |  |
| mmy3879 | Mat**α** | pob3-7::kanMX4 set1∆::natMX6 |  |
| mmy3880 | Mat**a** | pob3-7::kanMX4 jhd2∆::hygMX6 |  |
| mmy3897 | Mat**α** | pob3-L78R::kanMX4 jhd2∆::hygMX6 |  |
| mmy3898 | Mat**α** | pob3-L78R::kanMX4 |  |
| mmy3899 | Mat**α** | pob3-L78R::kanMX4 set1∆::natMX6 |  |
| mmy3900 | Mat**a** | pob3-L78R::kanMX4 set1∆::natMX6 jhd2∆::hygMX6 |  |
| mmy3901 | Mat**α** | sen1-1::kanMX4 set1∆::natMX6 jhd2∆::hygMX6 |  |
| mmy3902 | Mat**α** | sen1-1::kanMX4 set1∆::natMX6 |  |
| mmy3903 | Mat**a** | sen1-1::kanMX4 jhd2∆::hygMX6 |  |
| mmy3904 | Mat**α** | sen1-1::kanMX4 |  |
| mmy3962 | Mat**α** | nab3-11::kanMX4 set1∆::natMX6 jhd2∆::hygMX6 |  |
| mmy3963 | Mat**α** | nab3-11::kanMX4 set1∆::natMX6 |  |
| mmy4065 | Mat**α** | csf1∆::kanMX4 |  |
| mmy4067 | Mat**a** | csf1∆::kanMX4 jhd2∆::hygMX6 |  |
| mmy4166 | Mat**a** | GAL-JHD2(H427A)::natMX6 |  |
| mmy4167 | Mat**α** | csf1∆::kanMX4 GAL-JHD2(H427A)::natMX6 |  |
| mmy4170 | Mat**α** | csf1∆::kanMX4 GAL-JHD2::natMX6 |  |
| mmy4174 | Mat**α** | GAL-JHD2::natMX6 |  |
| mmy5364 | Mat**a**/**α** | trp1/+ csf1∆::kanMX4/+ hht1-hhf1∆::natMX4/+ hht2-hhf2::[HHTS-HHFS]H3K4R-URA3/+ set1∆::hygMX6/+ |  |
| mmy5365 | Mat**a**/**α** | met15/+ lys2/+ trp1/+ csf1∆::kanMX4/+ hht1-hhf1∆::natMX4/+ hht2-hhf2::[HHTS-HHFS]H3K4A-URA3/+ set1∆::hygMX6/+ |  |
| mmy6138 | Mat**a** | hht1-hhf1∆::natMX4 hht2-hhf2::[HHTS-HHFS]H3K4R-URA3 |  |
| mmy6140 | Mat**α** | hht1-hhf1∆::natMX4 hht2-hhf2::[HHTS-HHFS]H3K4R-URA3 set1∆::hygMX6 |  |
| mmy6187 | Mat**α** | nab3-11::kanMX4 hht1-hhf1∆::natMX4 hht2-hhf2::[HHTS-HHFS]H3K4WT-URA3 set1∆::hygMX6 |  |
| mmy6188 | Mat**α** | nab3-11::kanMX4 hht1-hhf1∆::natMX4 hht2-hhf2::[HHTS-HHFS]H3K4WT-URA3 |  |
| mmy6190 | Mat**a** | nab3-11::kanMX4 hht1-hhf1∆::natMX4 hht2-hhf2::[HHTS-HHFS]H3K4R-URA3 |  |
| mmy6191 | Mat**α** | nab3-11::kanMX4 hht1-hhf1∆::natMX4 hht2-hhf2::[HHTS-HHFS]H3K4R-URA3 jhd2∆::hygMX6 |  |
| mmy6210 | Mat**a** | hht1-hhf1∆::natMX4 hht2-hhf2::[HHTS-HHFS]H3K4A-URA3 |  |
| mmy6211 | Mat**α** | nab3-11::kanMX4 hht1-hhf1∆::natMX4 hht2-hhf2::[HHTS-HHFS]H3K4A-URA3 |  |
| mmy6212 | Mat**a** | nab3-11::kanMX4 hht1-hhf1∆::natMX4 hht2-hhf2::[HHTS-HHFS]H3K4A-URA3 set1∆::hygMX6 |  |
| mmy6213 | Mat**a** | hht1-hhf1∆::natMX4 hht2-hhf2::[HHTS-HHFS]H3K4A-URA3 set1∆::hygMX6 |  |
| mmy6219 | Mat**a** | hht1-hhf1∆::natMX4 hht2-hhf2::[HHTS-HHFS]H3K4WT-URA3 |  |
| mmy6220 | Mat**α** | hht1-hhf1∆::natMX4 hht2-hhf2::[HHTS-HHFS]H3K4WT-URA3 set1∆::hygMX6 |  |
| mmy6221 | Mat**a** | nab3-11::kanMX4 hht1-hhf1∆::natMX4 hht2-hhf2::[HHTS-HHFS]H3K4WT-URA3 |  |
| mmy6222 | Mat**α** | nab3-11::kanMX4 hht1-hhf1∆::natMX4 hht2-hhf2::[HHTS-HHFS]H3K4WT-URA3 |  |
| mmy6223 | Mat**a** | nab3-11::kanMX4 hht1-hhf1∆::natMX4 hht2-hhf2::[HHTS-HHFS]H3K4WT-URA3 jhd2∆::hygMX6 |  |
| mmy6224 | Mat**α** | nab3-11::kanMX4 hht1-hhf1∆::natMX4 hht2-hhf2::[HHTS-HHFS]H3K4WT-URA3 jhd2∆::hygMX6 |  |
| mmy6370 | Mat**a** | sen1-1::kanMX4 hht1-hhf1∆::natMX4 hht2-hhf2::[HHTS-HHFS]H3K4WT-URA3 |  |
| mmy6371 | Mat**α** | sen1-1::kanMX4 hht1-hhf1∆::natMX4 hht2-hhf2::[HHTS-HHFS]H3K4WT-URA3 set1∆::hygMX6 |  |
| mmy6384 | Mat**α** | sen1-1::kanMX4 hht1-hhf1∆::natMX4 hht2-hhf2::[HHTS-HHFS]H3K4R-URA3 |  |
| mmy6385 | Mat**α** | sen1-1::kanMX4 hht1-hhf1∆::natMX4 hht2-hhf2::[HHTS-HHFS]H3K4R-URA3 set1∆::hygMX6 |  |
| mmy6395 | Mat**α** | nab3-11::kanMX4 hht1-hhf1∆::natMX4 hht2-hhf2::[HHTS-HHFS]H3K4R-URA3 |  |
| mmy6396 | Mat**a** | nab3-11::kanMX4 hht1-hhf1∆::natMX4 hht2-hhf2::[HHTS-HHFS]H3K4R-URA3 jhd2∆::hygMX6 |  |
| mmy7658 | Mat**a**/**α** | csf1∆::kanMX4/+ hht1-hhf1∆::natMX4/hht1-hhf1∆::natMX4 hht2-hhf2::[HHTS-HHFS]H3K4R-URA3/+ set1∆::hygMX6/+ |  |
| mmy7659 | Mat**a**/**α** | csf1∆::kanMX4/+ hht1-hhf1∆::natMX4/hht1-hhf1∆::natMX4 hht2-hhf2::[HHTS-HHFS]H3K4WT-URA3/+ set1∆::hygMX6/+ |  |
| mmy7660 | Mat**a**/**α** | csf1∆::kanMX4/+ hht1-hhf1∆::natMX4/hht1-hhf1∆::natMX4 hht2-hhf2::[HHTS-HHFS]H3K4A-URA3/+ set1∆::hygMX6/+ |  |
| rjy068 | Mat**α** | spn1-K192N::kanMX4 jhd2∆::hygMX6 pDPM4-PYK1p-JHD2-FLAG |  |
| rjy069 | Mat**α** | spn1-K192N::kanMX4 jhd2∆::hygMX6 pDPM5-PYK1p-JHD2(H427A)-FLAG |  |
| rjy070 | Mat**α** | spn1-K192N::kanMX4 jhd2∆::hygMX6 pDPM6-PYK1p-JHD2(∆PHD)-FLAG |  |
| rjy071 | Mat**α** | spn1-K192N::kanMX4 jhd2∆::hygMX6 pDPM1-PYK1p-FLAG |  |
| rjy072 | Mat**α** | spt6-14::kanMX4 jhd2∆::hygMX6 pDPM4-PYK1p-JHD2-FLAG |  |
| rjy073 | Mat**α** | spt6-14::kanMX4 jhd2∆::hygMX6 pDPM5-PYK1p-JHD2(H427A)-FLAG |  |
| rjy074 | Mat**α** | spt6-14::kanMX4 jhd2∆::hygMX6 pDPM6-PYK1p-JHD2(∆PHD)-FLAG |  |
| rjy075 | Mat**α** | spt6-14::kanMX4 jhd2∆::hygMX6 pDPM1-PYK1p-FLAG |  |
| rjy219 | Mat**α** | spn1-K192N::kanMX4 pDPM1-PYK1p-FLAG |  |
| rjy327 | Mat**α** | nab3-42::URA3 |  |
|  |  |  |  |
|  |  |  |  |
|  |  |  |  |
|  |  |  |  |
|  |  |  |  |
|  |  |  |  |
