# Supplementary material for: H3K4 Methylation Dependent and Independent Chromatin Regulation by JHD2 and SET1 in Budding Yeast
Source: G3 (Bethesda). 2018 Mar 29;8(5):1829–39. doi: 10.1534/g3.118.200151 (PMC5940172; doi:10.1534/g3.118.200151)
Supplement: Supplementary file 8 [file 1829TableS2.docx]

| Name | Forward Primer | Reverse Primer |
| --- | --- | --- |
| SER3-1 | CGGTACCAACCAAGTTGACTTAGAC | ATTTCAGCGATGACCAATTCTGCTAC |
| SER3-3 | ACTAGATTAACTTCAAATGTCTTACAACATG | TGGTAGCGTAGTCTAAGTCAAC |
| SER3-4 | GACGTTCATGCTATTGGTATCAGATC | TACCGATACAGAAACAACCAATACAA |
| SER3-5 | TGCCCGAGGAAGAGTTGATC | CAAACCAGATTCTTCGCATGTTGTAA |
| SER3-6 | GAAGAGCAAGGTTACCAAGTCGAAT | CTAGTCTTTGATCTGATACCAATAGCATGAAC |
| SER3-7 | AAACGTTAATCAAACTGCTATTACAATCTT | GATCTTTTCGATCAACTCTTCCTCGG |
| SER3-8 | GCCTTTCTCAACGGGTGATATG | CAATGAAGATTTATAGAATTCGACTTGGTAAC |
| SER3-9 | ATGCTGTAAAGCACCCAAAAATTT | CGAAGATTGTAATAGCAGTTTGATTAACG |
| SER3-10 | CATGAATACCGTTCCACAGCG | ACGTTTTCTAATAGTAAAATCTTCATATCACC |
| SER3-11 | CCCAGGCGCTGTTTGTACTT | AAAGGCTTCAAAATTTTTGGGTG |
| SER3-12 | ACCTTTCAACAAGCTATGAATATGAGC | ACAGCATTCAAGCGCTGTGGA |
| SER3-13 | AATGACAAGCATTGACATTAACAACTTAC | CATGAAAGATTGCGTAGGTGAAGTAC |
| SER3-14 | TACAGAACTCTATAAAGAACCACAGAAAAATC | AGCCGCTCATATTCATAGCTTGTTG |
| SER3-16 | GGAAGAACCATTTCTAGTTATTTCACTTTT | CATTGCTGTCGATTTTTCTGTGGTTC |
| SER3-17 | GCAGAGGATAAGGAAATTCTTAAAACTG | GTTCTGTATTTTTACTAAGATAGTTGACAAG |
| SER3-19 | GGATGAAAAAATCAGACAAATATCCAA | CCTTTATATACATAACAGTTTTAAGAATTTCC |
| SER3-20 | TTAAGAAAATGCAACGCTGCC | GCTCCCTCCTTCCAACAAAG |
| SER3-22 | ATTCTTCTCGTTCCCACCTAATTTC | TCAGAAAACCCTGCACGGG |
| SER3-23 | GGAACAACTTCGGTCTCAGCA | TTTCTTAATTTTTTTCTCGTGGTAGAAG |
| SER3-24-2 | CGATATTTACTCACAAATGGAATTCAAG | GAAGTCAAGGACAATAAATTGCGAA |
| SER3-25-2 | AAACCTAATTTTTTTTGTGGACCCA | AACGAGAAGAATAATTAAAGTGCTGAGAC |
| SER3-26 | TAAAAATTTGGTTAAGCAGTTAGGCTG | TCCCCTTGAATTCCATTTGTGAGTAAATAT |
| SER3-27 | GCCAAGCTATGTGCAAATATCACAAA | TGGGTCCACAAAAAAAATTAGGTT |
| SER3-28 | CATTGTTTTAGTTTTTTACTCACAATCGA | AGGTCCAGCCTAACTGCTTA |
| SER3-29 | AGAAATGCCATTGTTTAATCCTGATT | TTTAATTTGTGATATTTGCACATAGCTTGG |
| SER3-31 | CATCTCCACCTTTCTCCCCAT | ACAATGTAGATAATCAGGATTAAACAATGGC |
| SER3-34 | TGCTGGATTGGATATATTGATAACGT | ATGTATCTCCACCTATCGTAAATTTGAAAG |
| SER3-35 | TCCATTTACTAATCAACTTAACAATGCTG | GTTCCGCTTTTCCGCCAAT |
| SER3-36 | TAAAACCCTTTTTTGTACACAATGGA | CAAACATCACGCAACGCTTTTT |
| SER3-37-2 | TATAACAAAATAATCAAGTTAAAACCCT | CGTTATCAATATATCCAATCCAGCATT |
| SER3-38-2 | TTCTTTACCTCATTCAACTGTATAGAACGT | GTTAAGTTGATTAGTAAATGGAAGAGATTCC |
| SER3-40-2 | GAGACTACACCGTGAAGCAACCT | AACGTTCTATACAGTTGAATGAGGTAAAGA |
| SER3-41 | TGATCAACTATTAAATTCCGGCAGTA | TTTAGTATAGATTATTTGGTAGCTTCAGG |
|  |  |  |
| FOR RT-PCR |  |  |
| SCR1_38F | AGAATTCTGGCCGAGGAAC |  |
| SCR1_151R | GAAAGACGGTTGCCACAAC |  |
| SER3_43F | GCGCTTGAATGCTGTAAAGC |  |
| SER3_403R | ACTCTTCCTCGGGCAATGAA |  |
